# Readdressing rapid sequence induction and intubation using ketamine or etomidate: A systematic review and meta-analysis of randomized clinical trials

**DOI:** 10.1097/MD.0000000000042207

**Published:** 2025-05-09

**Authors:** Lucas Bittar de Morais, Guilherme Requião Radel-Neto, Victor Alexandre dos Santos Valsecchi, Renan Alecsander Costa, Whady Hueb

**Affiliations:** a Universidade Anhembi Morumbi, São Paulo, Brazil; b Escola Paulista de Medicina, Federal University of São Paulo (EPM-UNIFESP), São Paulo, Brazil; c Heart Institute (InCor), Hospital das Clínicas, University of São Paulo Medical School, São Paulo, Brazil.

**Keywords:** etomidate, ketamine, RSII

## Abstract

**Background:**

The objective of this article is to clinically compare the inducing drugs ketamine and etomidate during the orotracheal intubation procedure in critically ill patients, aiming to reduce early mortality and other important complications involved in this act.

**Methods:**

This study is compliant to the PRISMA guidelines for systematic review and meta-analysis. A sensitive search was conducted using the databases PubMed (MEDLINE), Scopus, Lilacs (BVS), and Cochrane Library (Central). Our protocol included only randomized clinical trials, from the inception of the databases up to June 2024. Studies were selected if they compared ketamine to etomidate specifically for rapid sequence induction and intubation in critically ill patients. The outcomes assessed were: (1) all-cause mortality; (2) post-intubation arterial hypotension; (3) use of vasoactive drugs post-intubation; and (4) the incidence of adrenal insufficiency in the patient groups.

**Results:**

With the sensitive search strategy in question, we have identified 956 studies. Among these, 10 randomized clinical trials met the inclusion criteria, collectively involving a total of 2862 patients. Ketamine demonstrated comparable effectiveness to etomidate in preventing all-cause mortality (odds ratio [OR] = 0.8; 95% confidence interval [CI]: 0.65–1.21; *P* = .06). The rates of arterial hypotension post-intubation were also similar between the groups (OR = 1.28; 95% CI: 0.96–1.7; *P* = .34) and the same could be found when comparing the use of vasoactive drugs post-intubation (OR = 0.68; 95% CI: 0.36–1.27; *P* = .001). However, ketamine was less associated with adrenal insufficiency (OR = 0.35; 95% CI: 0.15–0.86; *P* = .008).

**Conclusion:**

Ketamine and etomidate demonstrated comparable effectiveness for rapid sequence intubation in terms of mortality and post-intubation hypotension. However, ketamine was associated with a lower risk of adrenal insufficiency, suggesting it may be a preferable option when patients are at high risk for adrenal suppression.

## 1. Introduction

Rapid sequence induction and intubation (RSII) is a critical procedure in emergency medicine and intensive care, requiring anesthetic agents that are both effective and safe. Ketamine and etomidate are among the most commonly used agents, as they provide optimal conditions for swift tracheal intubation.

Previous systematic reviews and meta-analyses have evaluated the effectiveness of these drugs in preventing mortality, hypotension, and other relevant outcomes during RSII.^[1,2]^ Ketamine is known for its anesthetic properties and its ability to maintain a stable cardiovascular profile, due to its stimulation of the central nervous system, increased heart rate, elevated cardiac output, and raised systolic blood pressure. In contrast, etomidate is favored for its capacity to preserve hemodynamic stability.^[3]^ Mechanistically, ketamine acts as an N-methyl-d-aspartate receptor antagonist, while etomidate functions as a gamma-aminobutyric acid type A receptor agonist.^[4]^

However, both agents are associated with distinct adverse effects. Ketamine is commonly linked to hallucinations, delirium, and elevated intraocular pressure, while etomidate is associated with adrenal suppression and metabolic disturbances.^[5]^

Despite their widespread use, uncertainties remain regarding the direct comparison of ketamine and etomidate, particularly in specific populations (e.g., elderly, pediatric, and individuals with chronic diseases).^[6]^ Key concerns include optimal dosing to minimize side effects, their efficacy and safety in these populations, and long-term clinical outcomes such as mortality, morbidity, and quality of life. These uncertainties are further compounded by the diverse settings in which RSII is performed, including prehospital emergencies, surgical theaters, and intensive care units.^[7–9]^

Therefore it is possible to hypothesize that ketamine may be a better drug for the RSII procedure in critically ill patients.

This article presents a systematic review and meta-analysis that clinically compares ketamine and etomidate in terms of their efficacy and safety for use in rapid sequence intubation.

## 2. Methods

This study is compliant to the PRISMA guidelines for systematic review and meta-analysis.^[10]^

Rapid sequence intubation was defined as the administration of an induction agent immediately followed by a neuromuscular blocking agent to achieve rapid unconsciousness and paralysis, followed by traqueal intubation.

### 2.1. Criteria for considering studies for this review

#### 2.1.1. Types of studies

We included all randomized controlled trials (RCTs) published in any language. Studies published in abstract form were also considered if they provided sufficient information regarding their methods and results. When necessary, we contacted the primary authors for additional details.

#### 2.1.2. Types of participants

Inclusion criteria: This review focused on critically ill patients who were at risk of death or faced imminent mortality and urgently required a definitive airway. Only adult participants (≥18 years old) were included, and only randomized clinical trials were eligible.

Exclusion criteria: We excluded non-randomized studies, patients who were already intubated, and those who were not in critical condition.

#### 2.1.3. Types of interventions

We considered the following comparison groups: rapid sequence induction and intubation using ketamine or its admixtures with those using etomidate.

#### 2.1.4. Types of outcome measures

We considered all outcome measures reported in the primary studies, accepting the definitions provided by the study authors. Where applicable, we discussed limitations, such as the use of non-validated instruments or inconsistent definitions.

#### 2.1.5. Primary outcomes

All-cause mortality (measured as time to death or frequency of deaths at any time point: in hospital, intensive care unit, or after discharge) and post-intubation hypotension as a dichotomous outcome (occurrence vs nonoccurrence).

#### 2.1.6. Secondary outcomes

Use of vasoactive drugs post-intubation and adrenal gland failure.

### 2.2. Search strategies for identifying studies

#### 2.2.1. Electronic searches

We have searched the following databases: Cochrane Central Register of Controlled Trials (CENTRAL) (The Cochrane Library 2024), MEDLINE (via PubMed) (inception to June 2024), EMBASE (via Ovid) (inception to June 2024), and LILACS (inception to June 2024).

The search strategy for MEDLINE included terms for clinical conditions, interventions, and their synonyms. This strategy was adapted as necessary for other databases. For MEDLINE, EMBASE, and LILACS, we used a highly sensitive filter for randomized controlled trials to optimize the search process. No language restrictions were applied.

See the complete search strategy located at Supplement 1, Supplemental Digital Content, http://links.lww.com/MD/O691.

#### 2.2.2. Other search methods

We manually searched the references of relevant articles, including other systematic reviews and RCTs in the area of rapid sequence intubation, and also ongoing RCTs in the Current Controlled Trials database at http://www.controlled-trials.com/.

#### 2.2.3. Data collection and analysis

**Figure 1.**
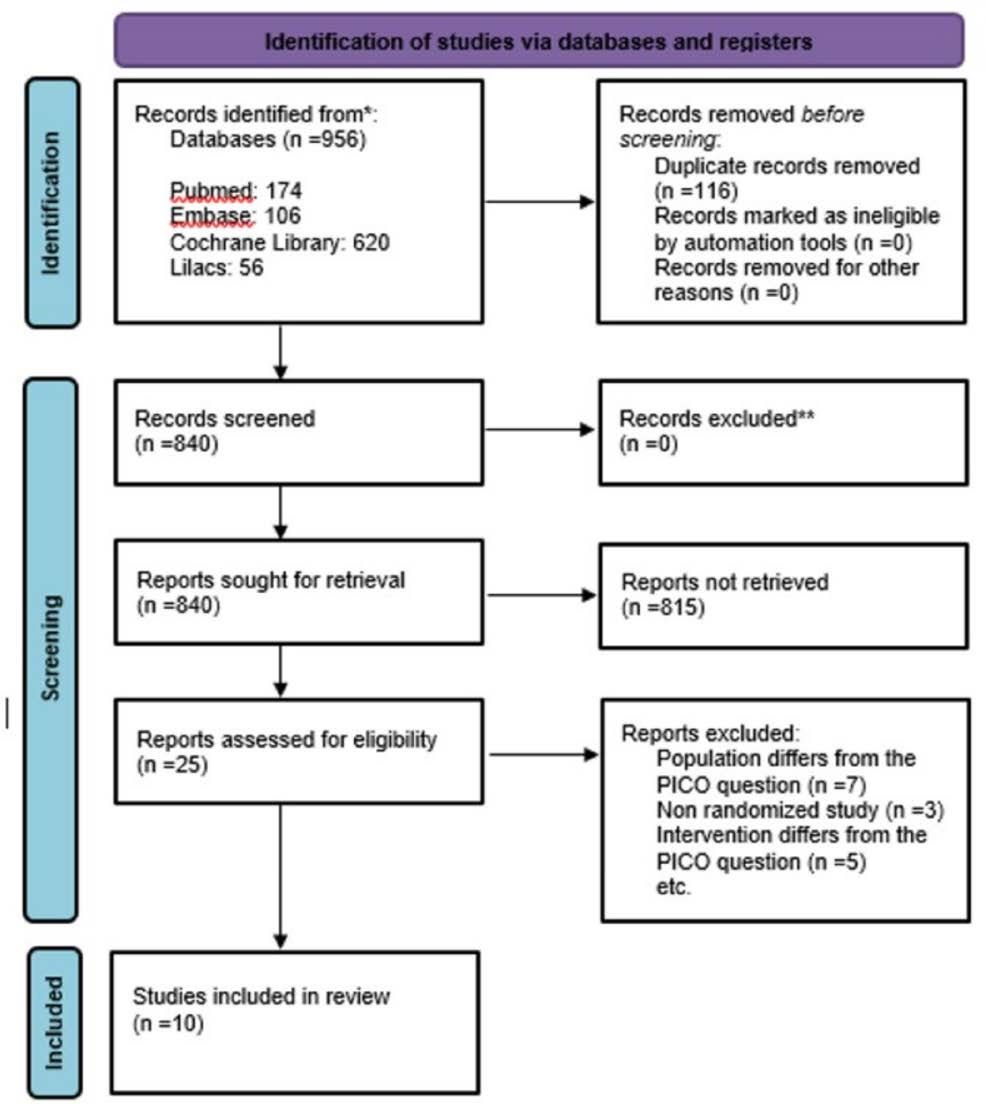
PRISMA flowchart of the study selection process. PRISMA = preferred reporting items for systematic reviews and meta-analyses.

**Figure 2.**
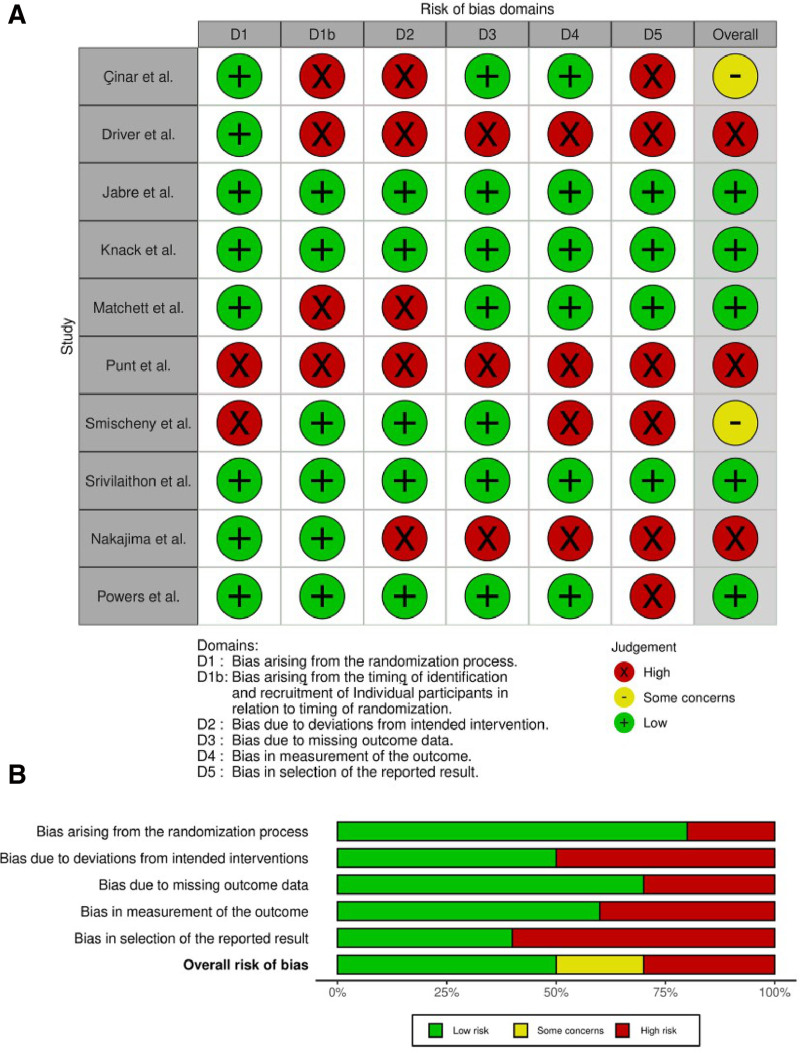
(A) RoB2 tool for risk of bias assessment traffic light plot. (B) RoB2 tool for risk of bias assessment graph (bar chart).

Selection of studies: Two authors (LBM and RAC) independently reviewed the titles and abstracts retrieved from the search (Fig. 1). Studies meeting the inclusion criteria were obtained in full text by GRRN and VV (Fig. 2).

Data extraction and management: Two reviewers (LBM and RAC) extracted data using the Rayyan platform, including study design, participant characteristics, interventions (e.g., intubation procedures and materials), and results. Discrepancies were resolved by consensus. Authors of primary studies were contacted for additional information when necessary. Later, GRRN and LBM abstracted data into Review Manager (RevMan 5.4), and a third author (VV) rechecked entries for accuracy.

#### 2.2.4. Assessment of risk of bias

Two reviewers (GRRN and VV) assessed the methodological quality of studies based on the criteria in the Cochrane Handbook for Systematic Reviews of Interventions.^[11]^ The following criteria were evaluated:

- Adequate generation of the random allocation sequence.
- Adequate concealment of allocation.
- Adequate prevention of knowledge about interventions for data collectors (or independence of data collectors from study planners).
- Addressing incomplete outcome data.
- Avoidance of selective reporting.

Each item was classified as having a low, high, or unclear risk of bias. Blinding was considered only at the data collection level due to the nature of the interventions.

#### 2.2.5. Measures of treatment effect

For comparable studies, dichotomous data were expressed as odds ratios (OR) and to continuous data, the effect measure selected was mean difference, both with 95% confidence intervals (CI) and using a random-effects model.

#### 2.2.6. Unit of analysis issues

The unit of analysis was based on the individual patient (the unit randomized for intervention comparison). Cross-over designs were not expected due to the intervention’s nature.

#### 2.2.7. Dealing with missing data

Dropout rates were reported in the “Characteristics of Included Studies” table. Intention-to-treat analysis was performed for dichotomous data.

#### 2.2.8. Assessment of heterogeneity

We used a random-effects model for data presentation and quantified heterogeneity using the chi square statistic and *I*² statistic. The following thresholds guided interpretation:

- 0%–40%: May not be important.
- 30%–50%: May represent moderate heterogeneity.
- 50%–90%: May represent substantial heterogeneity.
- 75%–100%: Considerable heterogeneity.

#### 2.2.9. Assessment of reporting biases

We planned to assess publication bias using a funnel plot if enough studies were available.

#### 2.2.10. Data synthesis

Qualitative data (e.g., methods, risk of bias, and participant characteristics) were summarized in a table. Quantitative data were synthesized using a random-effects model. Nonparametric data or insufficiently detailed data were documented in an appendix.

#### 2.2.11. Sensitivity analysis

If sufficient studies were available, sensitivity analyses were planned to explore heterogeneity and result robustness, focusing on allocation concealment, blinding, statistical models, and intention-to-treat analysis. These findings were intended for hypothesis generation rather than conclusive evidence.

#### 2.2.12. Ethical review

Since this is a systematic review and meta-analysis, ethical review was not applicable.

## 3. Results

A total of 10 RCTs met the inclusion criteria and were included in this systematic review and meta-analysis. Their characteristics are shown in Table 1, as well as the averages of the overall population characteristics on their clinically relevant characteristics, which are given in Table 2.

**Table 1:** A summary of the general characteristics of the included studies.

Study ID	Total sample	Ketamine	Etomidate	Blinding	No. of included centers	Follow-up duration (d)
Knack et al^[6]^	143	70	73	Blinded	Single-center	30
Jabre et al^[7]^	655	327	328	Blinded	Multicentric	28
Smischney et al^[8]^	160	79	73	Open label	Single-center	30
Çinar et al^[12]^	22	10	12	Open label	Single-center	30
Driver et al^[12]^	54	26	28	Open label	Single-center	28
Powers et al^[12]^	398	204	194	Open label	Single-center	30
Punt et al^[13]^	301	140	161	Open label	Single-center	28
Nakajima et al^[14]^	68	37	31	Open label	Single-center	60
Matchet et al^[15]^	801	400	401	Open label	Multicentric	28
Srivilaithon et al^[16]^	260	130	130	Blinded	Single-center	28

**Table 2:** Clinically relevant characteristics of the studies populations.

Baseline characteristics	Ketamine	Etomidate
Age, years, mean, SD	62.3 (7.5)	59.8 (7.5)
Male (%)	60	57.6
Weight (kg), mean, SD	77.3 (7)	77 (5.9)
Previous comorbidities (%)	Ketamine	Etomidate
Hypertension	48.6	52.3
Diabetes	29.5	25.3
CKD	8.5	11.8
Heart failure	16.6	11.9
Stroke	15.2	19.8
COPD	14	7.7
Smoking	22	19
Cancer	4.5	2.5
Reason for RSII (%)	Ketamine	Etomidate
Comastose	27	30
Hemodynamic shock	20	23
Acute respiratory failure	35	33
Other	18	14
Vital signs (SD)	Ketamine	Etomidate
Temperature (°C)	36.7 (0.2)	36.6 (0.1)
Heart rate (BPM)	103.5 (6.4)	104.8 (5.8)
Systolic blood pressure (mm Hg)	126.3 (8.1)	126.4 (10.3)
Diastolic blood pressure (mm Hg)	74.6 (0.4)	75.3 (2.6)
SpO2 (%)	95.7 (2.8)	95 (2.2)
GCS (median)	7 (7–9)	8 (6–9)

BPM = beats per minute, CKD = chronic kidney disease, COPD = chronic obstructive pulmonary disease, GCS = glasgow coma scale, SD = standard deviation.

Regarding the primary outcome of overall mortality, no statistically relevant difference was observed between the ketamine and etomidate groups (OR: 0.88; 95%CI: 0.64–1.21).

Similarly, for the outcome of post-intubation hypotension, with an OR of 1.28 (95% CI: 0.96–1.70), which also lacked statistical significance.

The use of vasoactive drugs post-intubation comparison yielded an OR of 0.68 (95% CI: 0.36–1.27), favoring ketamine, though the result was not statistically significant.

However, adrenal failure was more prevalent in the etomidate group, demonstrating a statistically significant difference, with ketamine associated with a lower risk (OR: 0.35; 95% CI: 0.15–0.83), highlighting an advantage of the latter in preserving adrenal function.

**Figure 3.**
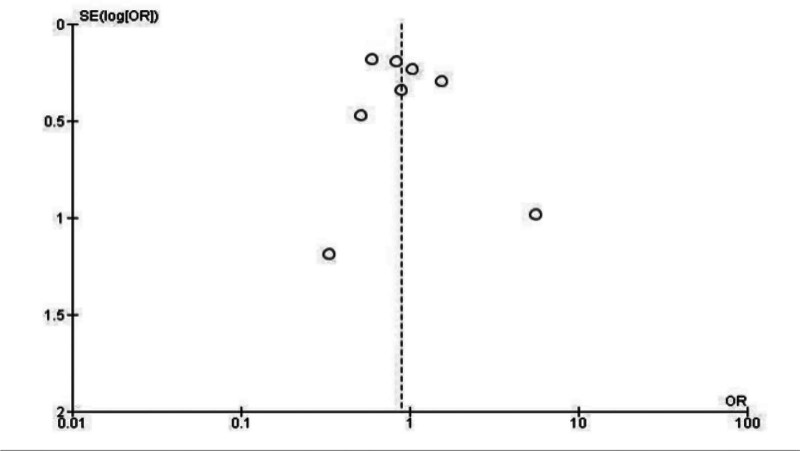
Funnel plot evaluating the outcome of all-cause mortality.

**Figure 4.**
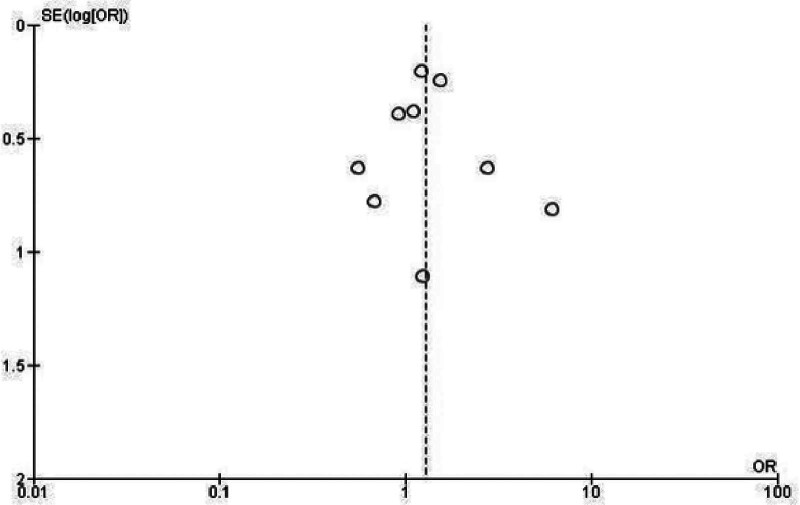
Funnel plot evaluating the outcome of post-intubation hypotension.

To assess the consistency and potential publication bias of the studies included, funnel plots were generated for each of the outcomes analyzed, when feasible. These plots provided a visual representation of the distribution of effect sizes across studies, ensuring reliability in the findings. Thus, at visual inspection, these funnel plots show a low risk of publication bias presented by this review. Because the larger studies shown in the superior portion of the graphic are distributed symmetrically (Figs. 3 and 4).

## 4. Discussion

The findings of this analysis provide valuable insights into the benefits and limitations of these commonly used anesthetic agents (ketamine and etomidate), with important clinical implications for patient management in emergency and critical care settings.

### 4.1. Interpretation of findings

#### 4.1.1. All-cause mortality

**Figure 5.**
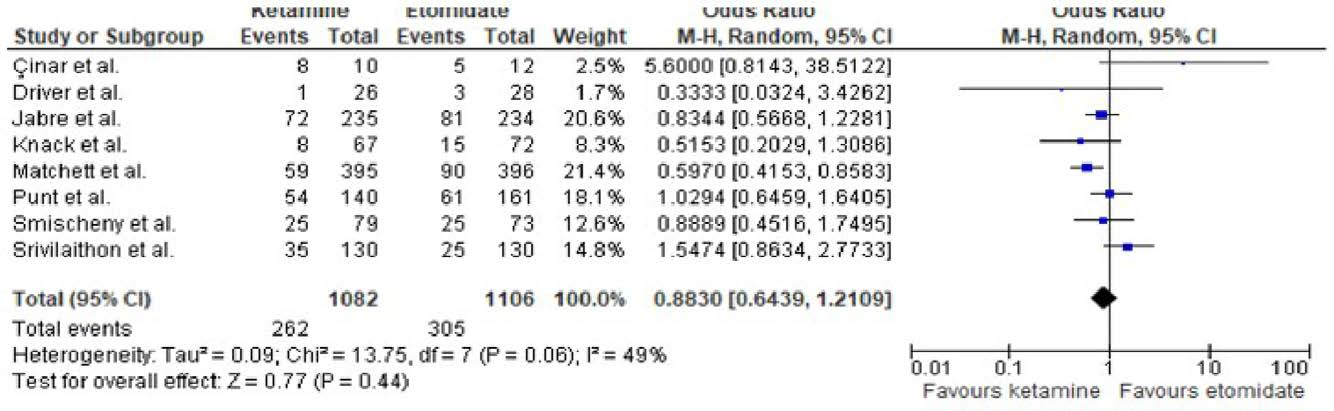
Forest plot representing the outcome of all-cause mortality.

The absence of a statistically significant difference in all-cause mortality between ketamine and etomidate (OR: 0.88; 95% CI: 0.64–1.21), shown in Figure 5, suggests that both agents are effective in supporting critically ill patients through RSII without impacting survival. This particular finding underscores their comparable utility in this high-stakes procedure, considering that most deaths in such scenarios would not happen, only as a consequence of this procedure.^[12,13]^ It is important to consider that such event is influenced by numerous factors beyond the choice of induction agent, such as underlying disease severity, the presence of comorbidities, and overall management strategies in the intensive care unit.

#### 4.1.2. Post-intubation hypotension

**Figure 6.**
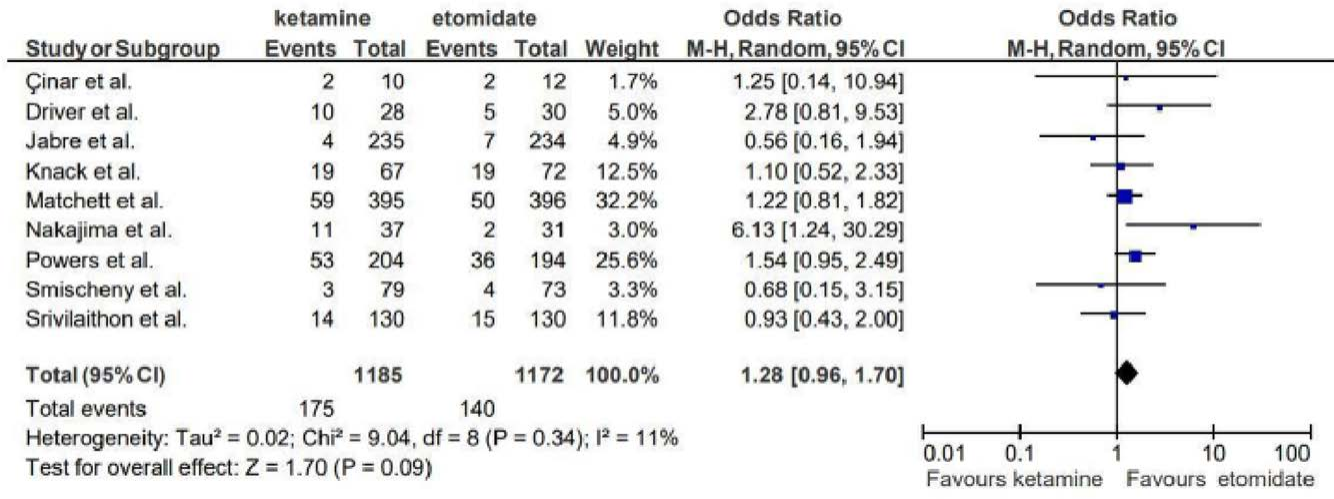
Forest plot representing the outcome of post-intubation hypotension.

The trend toward increased post-intubation hypotension with ketamine aligns with most of the current literature^[5]^ (OR: 1.28; 95% CI: 0.96–1.70), though not statistically significant, it may be justified by the pharmacological properties of the 2 drugs (Fig. 6). Ketamine’s cardiovascular-stimulating effects, including increased heart rate and cardiac output, apparently até still not enough to make it a preferred agent for hemodynamically unstable patients. This result may be particularly relevant in contexts such as septic shock, where maintaining blood pressure is critical for organ perfusion and patient outcomes.^[14]^

#### 4.1.3. Use of vasoactive drugs

**Figure 7.**
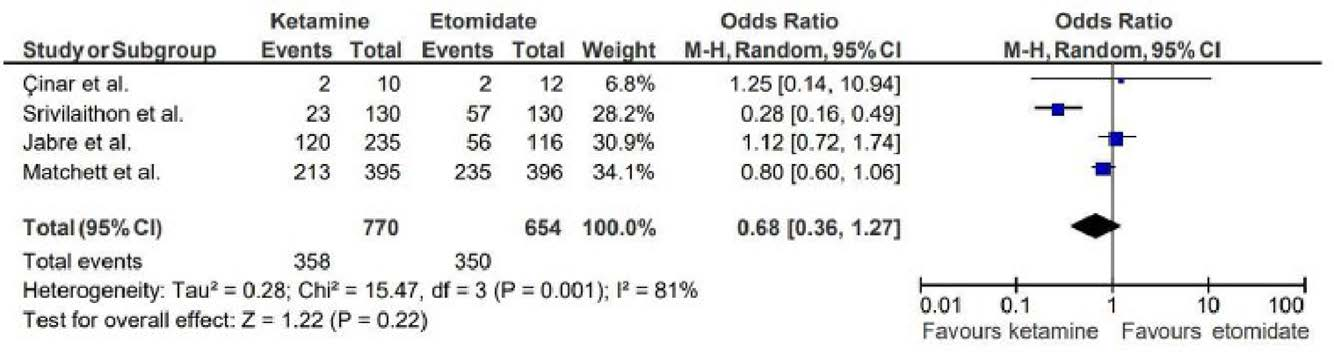
Forest plot representing use of vasoactive drugs.

Contradictorily, the reduced need for vasoactive drugs post-intubation in the ketamine group (OR: 0.68; 95% CI: 0.36–1.27) might be associated to its potential hemodynamic transitory advantages (Fig. 7). Although the result was not statistically significant, this trend could hold clinical relevance in resource-limited settings or situations where minimizing the use of additional medications is desirable. Further studies with larger sample sizes are needed to clarify whether this trend is consistent across different patient populations.^[5,16]^

#### 4.1.4. Adrenal failure

**Figure 8.**
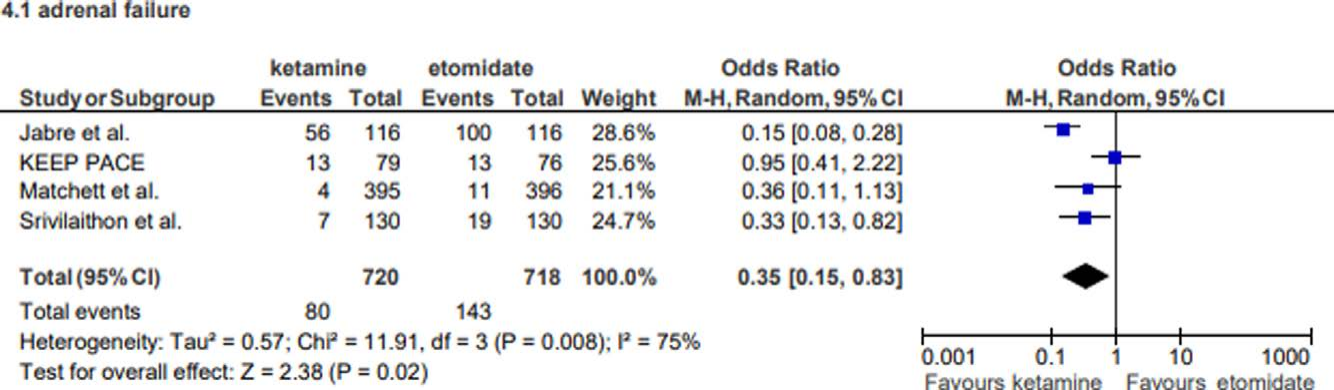
Forest plot representing the outcome of adrenal failure.

The significant reduction in adrenal failure associated with ketamine (OR: 0.35; 95% CI: 0.15–0.83) highlights an important limitation of etomidate. As a potent inhibitor of 11β-hydroxylase, etomidate can suppress adrenal steroidogenesis, leading to adrenal insufficiency (Fig. 8). This effect is particularly concerning in critically ill patients, who often rely on adequate adrenal function to mount a stress response. The findings suggest that ketamine may be a safer choice in scenarios where adrenal function is already compromised, such as in sepsis or prolonged critical illness.^[15]^

#### 4.1.5. Broader implications

These results emphasize the need for personalized approaches to RSII, considering the unique physiological and clinical characteristics of each patient. While ketamine appears to offer advantages in terms of hemodynamic stability and reduced risk of adrenal failure, etomidate may still be appropriate in specific circumstances where these concerns are less relevant, such as in patients with stable hemodynamics or those undergoing brief procedures.

The use of funnel plots to assess publication bias further strengthens the reliability of these findings. By evaluating the consistency of results across studies, this analysis helps ensure that conclusions are not unduly influenced by selective reporting or small-study effects.

#### 4.1.6. Limitations

Several limitations of this review warrant discussion. The most important ones are:

Sample size and statistical power: While there was a numerical difference favoring ketamine observed in some outcomes, the lack of statistical significance in others, such as post-intubation hypotension and vasoactive drug use, may reflect insufficient power due to the limited number of included studies, as well as the small sample sizes in most of the selected trials.

Heterogeneity: Differences in patient populations, clinical settings, presence of other inductive drugs (admixtures), and dosing regimens across studies introduce variability that may have influenced the results.

Outcome definitions: Variability in how outcomes were defined and measured by the included studies may have affected the robustness of the findings. For example, thresholds for diagnosing adrenal failure or defining hypotension were not consistent.

Short-term focus: The analysis primarily focused on short-term outcomes, with limited information on long-term effects such as mortality beyond hospital discharge, functional recovery, and quality of life.

#### 4.1.7. Future research directions

Investigating the effects of ketamine and etomidate in specific subgroups, such as pediatric, elderly, and immunocompromised patients. As well as, standardizing outcome definitions and measurement methods to improve comparability across studies. Exploring long-term clinical outcomes, including survival, morbidity, and quality of life, to better understand the broader implications of these induction agents, should also be done. Conducting larger, multicenter trials to provide more robust evidence regarding the trends observed in this analysis is also necessary.

## 5. Conclusion

In the evaluated sample, ketamine and etomidate demonstrated comparable effectiveness for rapid sequence induction and intubation in terms of mortality and post-intubation hypotension. However, ketamine was associated with a lower risk of adrenal insufficiency, suggesting it may be a preferable option for patients at a high risk for adrenal suppression.

## Acknowledgments

We also would like to express our gratitude to the teams of the Cochrane Center in Brazil (EPM-UNIFESP) and the MASS research group (InCor-HCFMUSP).

## Author contributions

**Data curation:** Lucas Bittar de Morais, Guilherme Requião Radel-Neto.

**Formal analysis:** Guilherme Requião Radel-Neto, Victor Alexandre dos Santos Valsecchi.

**Funding acquisition:** Lucas Bittar de Morais.

**Methodology:** Lucas Bittar de Morais.

**Project administration:** Lucas Bittar de Morais, Guilherme Requião Radel-Neto.

**Resources:** Guilherme Requião Radel-Neto, Renan Alecsander Costa.

**Software:** Lucas Bittar de Morais, Guilherme Requião Radel-Neto, Victor Alexandre dos Santos Valsecchi.

**Supervision:** Whady Hueb.

**Validation:** Lucas Bittar de Morais, Victor Alexandre dos Santos Valsecchi.

**Visualization:** Guilherme Requião Radel-Neto, Renan Alecsander Costa.

**Writing—original draft:** Lucas Bittar de Morais.

**Writing—review & editing:** Guilherme Requião Radel-Neto, Victor Alexandre dos Santos Valsecchi.

## Supplementary Material



## References

[1] Koroki T Kotani Y Yaguchi T. Ketamine versus etomidate as an induction agent for tracheal intubation in critically ill adults: a Bayesian meta-analysis. Crit Care. 2024;28:48. 38368326 10.1186/s13054-024-04831-4 PMC10874027

[2] Sharda SC Bhatia MS. Etomidate compared to ketamine for induction during rapid sequence intubation: a systematic review and meta-analysis. Indian J Crit Care Med. 2022;26:108–13. 35110853 10.5005/jp-journals-10071-24086 PMC8783236

[3] Kunkel S Lenz T. Hemodynamics in helicopter emergency medical services (HEMS) patients undergoing rapid sequence intubation with etomidate or ketamine. J Emerg Med. 2022;62:163–70. 35031173 10.1016/j.jemermed.2021.10.004

[4] Fischell J Van Dyke A Kvarta M. Rapid antidepressant action and restoration of excitatory synaptic strength after chronic stress by negative modulators of alpha5-containing GABAA receptors. Neuropsychopharmacol. 2015;40:2499–509. 10.1038/npp.2015.112 PMC4569955 25900119

[5] Mohr NM Pape SG Runde D Kaji AH Walls RM Brown CA 3rd. Etomidate use is associated with less hypotension than ketamine for emergency department sepsis intubations: a NEAR cohort study. Acad Emerg Med. 2020;27:1140–9. 32602974 10.1111/acem.14070 PMC8711033

[6] Knack SKS Prekker ME Moore JC. The effect of ketamine versus etomidate for rapid sequence intubation on maximum sequential organ failure assessment score: a randomized clinical trial. J Emerg Med. 2023;65:e371–82. 37741737 10.1016/j.jemermed.2023.06.009

[7] Jabre P Combes X Lapostolle F. Etomidate versus ketamine for rapid sequence intubation in acutely ill patients: a multicentre randomised controlled trial. Lancet. 2009;374:293–300. 19573904 10.1016/S0140-6736(09)60949-1

[8] Smischney NJ Nicholson WT Brown DR. Ketamine/propofol admixture vs etomidate for intubation in the critically ill: KEEP PACE Randomized clinical trial. J Trauma Acute Care Surg. 2019;87:883–91. 31335755 10.1097/TA.0000000000002448

[9] Yoğun Bakim Hastalarinin Endotrakeal Entübasyonunda Ortaya Çikan Metabolik ve Hemodinamik Yanitlara Ketamin ve Etomidatin Etkilerinin Karşilaştirilmasi. Türk Yoğun Bakim Derneği Dergisi. 2011;9:77–84.

[10] Page MJ McKenzie JE Bossuyt PM. The PRISMA 2020 statement: an updated guideline for reporting systematic reviews. BMJ. 2021;372:n71. 33782057 10.1136/bmj.n71 PMC8005924

[11] Higgins JPT Thomas J Chandler J Cumpston M Li T Page MJ Welch VA (editors). *Cochrane Handbook for Systematic Reviews of Interventions* version 6.5 (updated August 2024). Cochrane; 2024. Available at: www.training.cochrane.org/handbook.

[12] Driver B Reardon R McGill J. ED airway management of severe angioedema: a single center’s experience. Acad Emerg Med. 2014;21(Suppl 1):S116. https://onlinelibrary.wiley.com/doi/10.1111/acem.12365.

[13] Punt CD Dormans TPJ Oosterhuis WP. Etomidate and S-ketamine for the intubation of patients on the intensive care unit: a prospective, open-label study. Neth J Crit Care. 2014;18:4–7.

[14] Nakajima S Taylor K Zimmerman LH Collopy K Fales C Powers W. 848: Hemodynamic effects of ketamine versus etomidate during rapid sequence intubation in an ed. Crit Care Med. 2019;47:403–403.30585789

[15] Matchett G Gasanova I Riccio CA. Etomidate versus ketamine for emergency endotracheal intubation: a randomized clinical trial. Intensive Care Med. 2022;48:78–91. 34904190 10.1007/s00134-021-06577-x

[16] Srivilaithon W Bumrungphanithaworn A Daorattanachai K. Clinical outcomes after a single induction dose of etomidate versus ketamine for emergency department sepsis intubation: a randomized controlled trial. Sci Rep. 2023;13:6362. 37076524 10.1038/s41598-023-33679-x PMC10115773

