# Supplementary material for: Readdressing rapid sequence induction and intubation using ketamine or etomidate: A systematic review and meta-analysis of randomized clinical trials
Source: Medicine (Baltimore). 2025 May 9;104(19):e42207. doi: 10.1097/MD.0000000000042207 (PMC12074070; doi:10.1097/MD.0000000000042207)

Supplementary material

Complete Search Strategy

#1 "Rapid Sequence Induction and Intubation"[MeSH Terms] OR (Rapid Sequence Induction and Intubation) OR (Rapid Sequence Intubation) OR (Rapid Sequence) OR (Induction) OR (Intubation, Rapid Sequence)

#2 "Intubation, Intratracheal"[Mesh] OR (Intratracheal Intubation) OR (Intratracheal Intubations) OR (Intubations, Intratracheal) OR (Intubation, Endotracheal) OR (Endotracheal Intubation) OR (Endotracheal Intubations) OR (Intubations, Endotracheal)

#3 "Etomidate"[MeSH Terms] OR (Hypnomidate) OR (R-26490) OR (R26490) OR (R 26490) OR (Radenarkon)

#4 "Ketamine"[MeSH Terms] OR (Ketamine) OR (2-(2-Chlorophenyl)-2-(methylamino)cyclohexanone) OR

(Ketalar) OR (CI-581) OR (CI581) OR (CI 581) OR (Ketanest) OR (Ketamine Hydrochloride) OR (Calipsol) OR (Calypsol) OR (Kalipsol) OR (Ketaset)

#5 #1 OR #2

#6 #3 AND #4

#7 5 AND #6

#8 ((clinical[Title/Abstract] AND trial[Title/Abstract]) OR clinical trials as topic[MeSH Terms] OR clinical trial[Publication Type] OR random*[Title/Abstract] OR random allocation[MeSH Terms] OR therapeutic use[MeSH Subheading])

#9 #7 AND #8


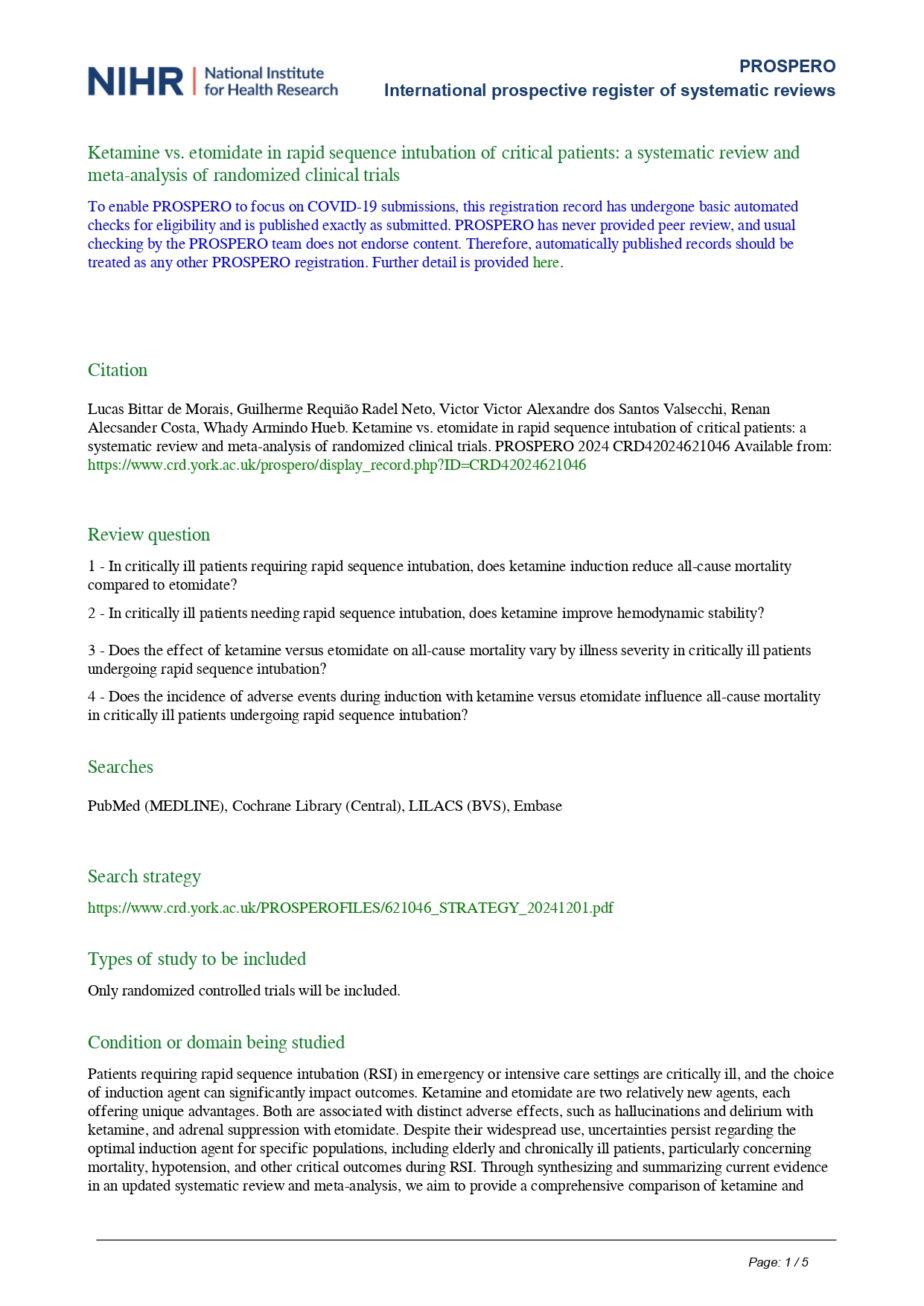


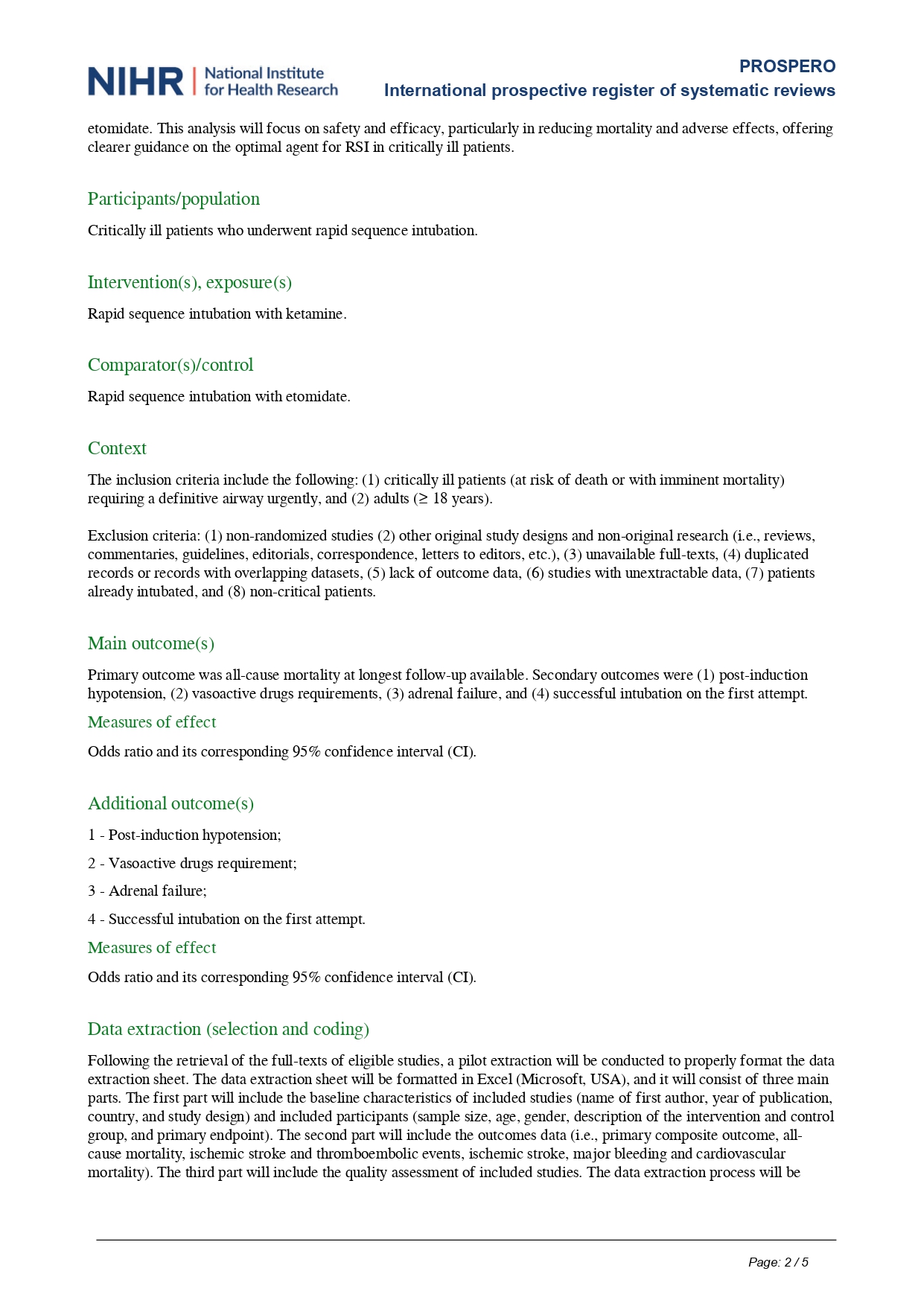


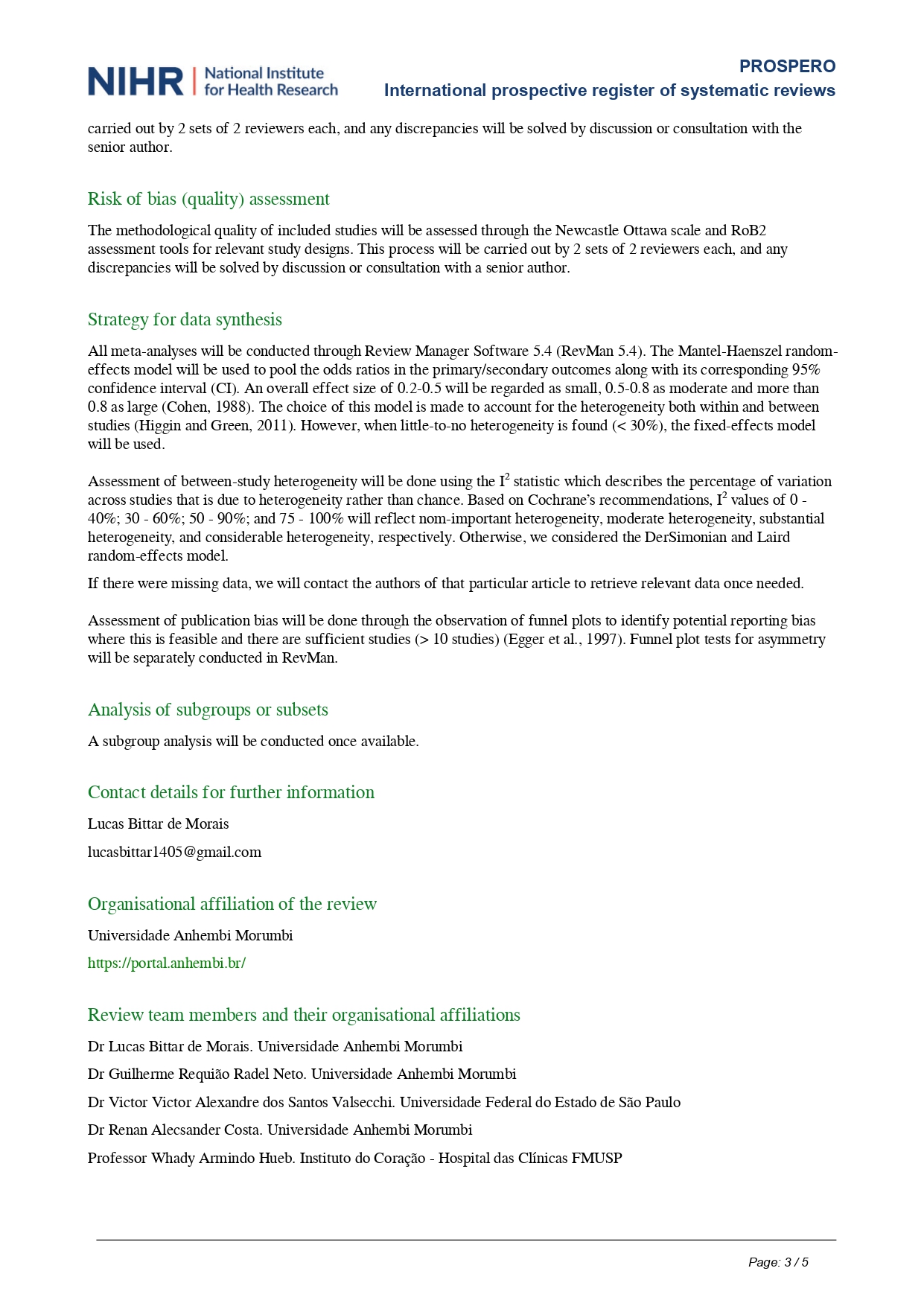


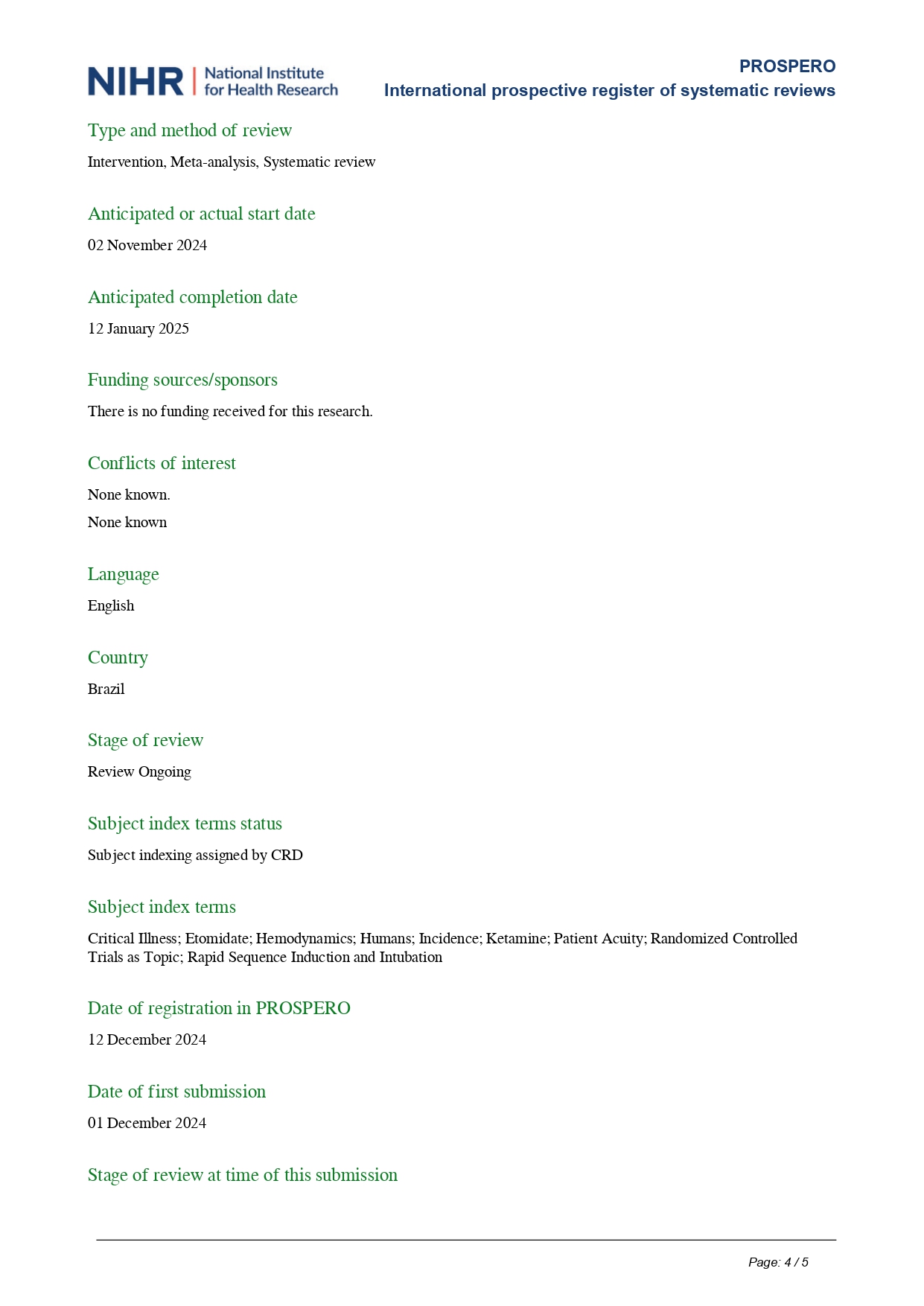


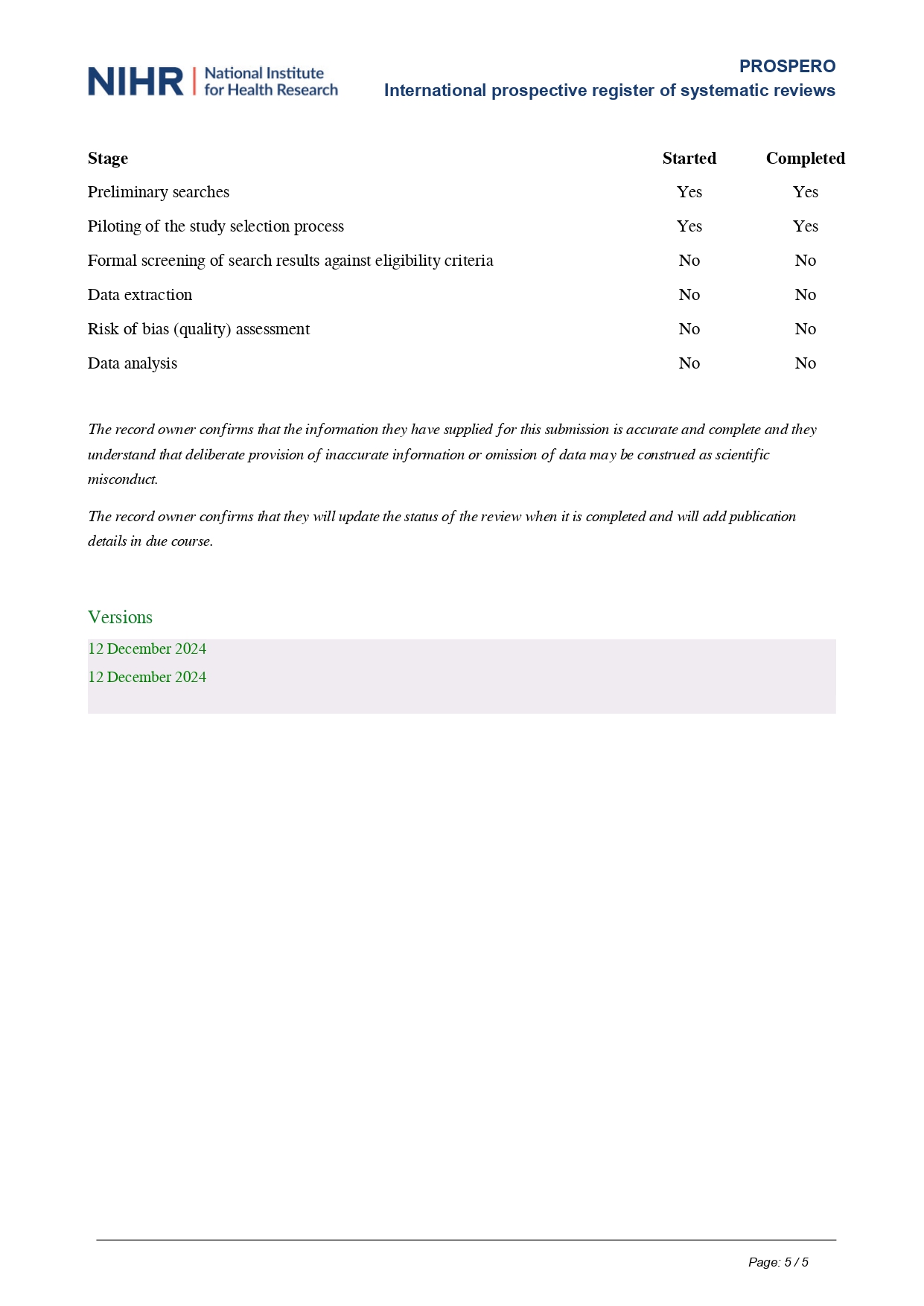

Supplement: Supplementary file 1 [file medi-104-e42207-s001.docx]
